# Atresia of the Transverse Colon (Grosfeld Type IIIa) Diagnosed at Seven Days of Life: A Case Report

**DOI:** 10.7759/cureus.113901

**Published:** 2026-08-03

**Authors:** Urias J Hernandez Lopez, Pastor A Thomas Olivares, Adriana P Redondo Rada, Vanessa P Quiñones Cantillo, Vicky G Gomez Montes

**Affiliations:** 1 Department of Surgery, Universidad del Sinú - Seccional Cartagena, Cartagena, COL; 2 Faculty of Medicine, Universidad del Sinú - Seccional Cartagena, Cartagena, COL; 3 Faculty of Medicine, Universidad del Magdalena, Santa Marta, COL

**Keywords:** colonic atresia, intestinal obstruction, newborn, primary anastomosis, right hemicolectomy, transverse colon

## Abstract

Colonic atresia is a rare cause of neonatal intestinal obstruction, and involvement of the transverse colon is particularly uncommon. We report a seven-day-old term male neonate with failure to pass meconium since birth, bilious vomiting, abdominal distension, and jaundice. Plain abdominal radiography demonstrated diffuse bowel-loop dilatation with relative paucity of distal gas. Exploratory laparotomy revealed atresia of the transverse colon (Grosfeld type IIIa), with a markedly dilated proximal colon, an atrophic distal colon, and a V-shaped mesenteric defect. A right hemicolectomy was performed, followed by a hand-sewn end-to-end anastomosis between the terminal ileum and the descending colon. During the postoperative course, late-onset neonatal sepsis was diagnosed, with *Enterobacter cloacae* detected by a molecular sepsis panel despite negative conventional blood cultures. The patient was successfully treated with cefepime, without anastomotic or intra-abdominal complications. Enteral feeding was progressively introduced, and the patient achieved full tolerance of breastfeeding and was discharged in good condition. This case emphasizes the importance of considering colonic atresia in neonates with persistent distal intestinal obstruction and illustrates that primary anastomosis may be feasible in selected patients when distal bowel patency and the clinical condition are favorable.

## Introduction

Intestinal atresias are congenital malformations characterized by complete interruption of gastrointestinal luminal continuity and represent an important cause of neonatal intestinal obstruction [1]. Colonic atresia is the rarest form of intestinal atresia, accounting for approximately 2-15% of cases, with an estimated prevalence ranging from 0.15 to one per 10,000 live births [2]. Involvement of the transverse colon is particularly uncommon.

Affected neonates typically present with failure to pass meconium, progressive abdominal distension, and bilious vomiting, consistent with distal intestinal obstruction. According to the modified Grosfeld classification, type I consists of an intraluminal mucosal web with continuity of the bowel wall and mesentery; type II comprises two blind-ending segments connected by a fibrous cord with an intact mesentery; type IIIa is characterized by complete separation of the atretic ends with a V-shaped mesenteric defect; type IIIb corresponds to the “apple-peel” deformity, with extensive mesenteric loss and the distal bowel spiraling around a single feeding vessel; and type IV comprises multiple intestinal atresias [3].

We report a seven-day-old neonate with intestinal obstruction caused by atresia of the transverse colon (Grosfeld type IIIa), managed with right hemicolectomy and a primary hand-sewn end-to-end anastomosis between the terminal ileum and the descending colon.

## Case presentation

Patient information and physical examination

A term male neonate born at 38 weeks of gestation after an uncomplicated pregnancy with adequate prenatal care was admitted at seven days of life because of failure to pass meconium since birth, bilious vomiting, abdominal distension, and jaundice. His birth weight was 2775 g. On admission, he was afebrile, jaundiced, and tachycardic, with a heart rate of 165 beats per minute, respiratory rate of 40 breaths per minute, oxygen saturation of 99%, a and blood pressure of 105/66 mmHg (mean arterial pressure: 79 mmHg). His weight was 2345 g, representing a 15.5% decrease from his birth weight. The abdomen was soft but distended, without signs of peritoneal irritation. The anus was patent, with no evidence of an anorectal malformation. Neurological examination and primitive neonatal reflexes were normal.

Diagnostic assessment and surgical treatment

Initial laboratory testing showed predominantly indirect hyperbilirubinemia, with a total bilirubin level of 14.62 mg/dL, direct bilirubin of 0.50 mg/dL, and indirect bilirubin of 14.12 mg/dL. The complete blood count, serum electrolyte levels, blood glucose, and C-reactive protein were within normal limits. Urinalysis showed no findings suggestive of infection, and urine culture showed no bacterial growth.

**Figure 1:**
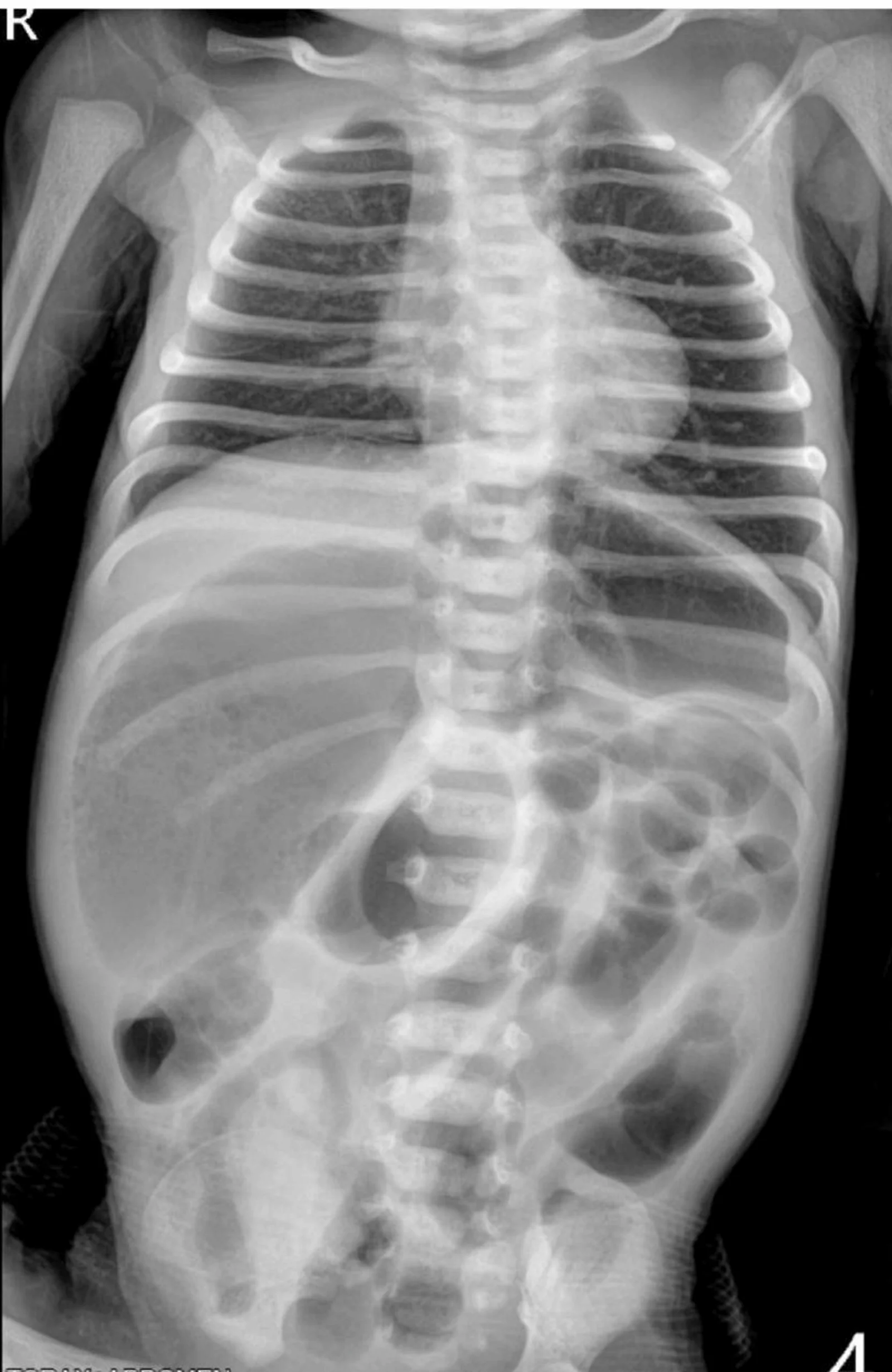
Abdominal radiograph Supine anteroposterior abdominal radiograph showing diffuse bowel-loop dilatation and relative paucity of distal gas, without evidence of pneumoperitoneum.

On admission, plain abdominal radiography demonstrated diffuse bowel-loop dilatation with relative paucity of distal gas and no evidence of pneumoperitoneum, suggesting a distal intestinal obstruction (Figure 1).

**Figure 2:**
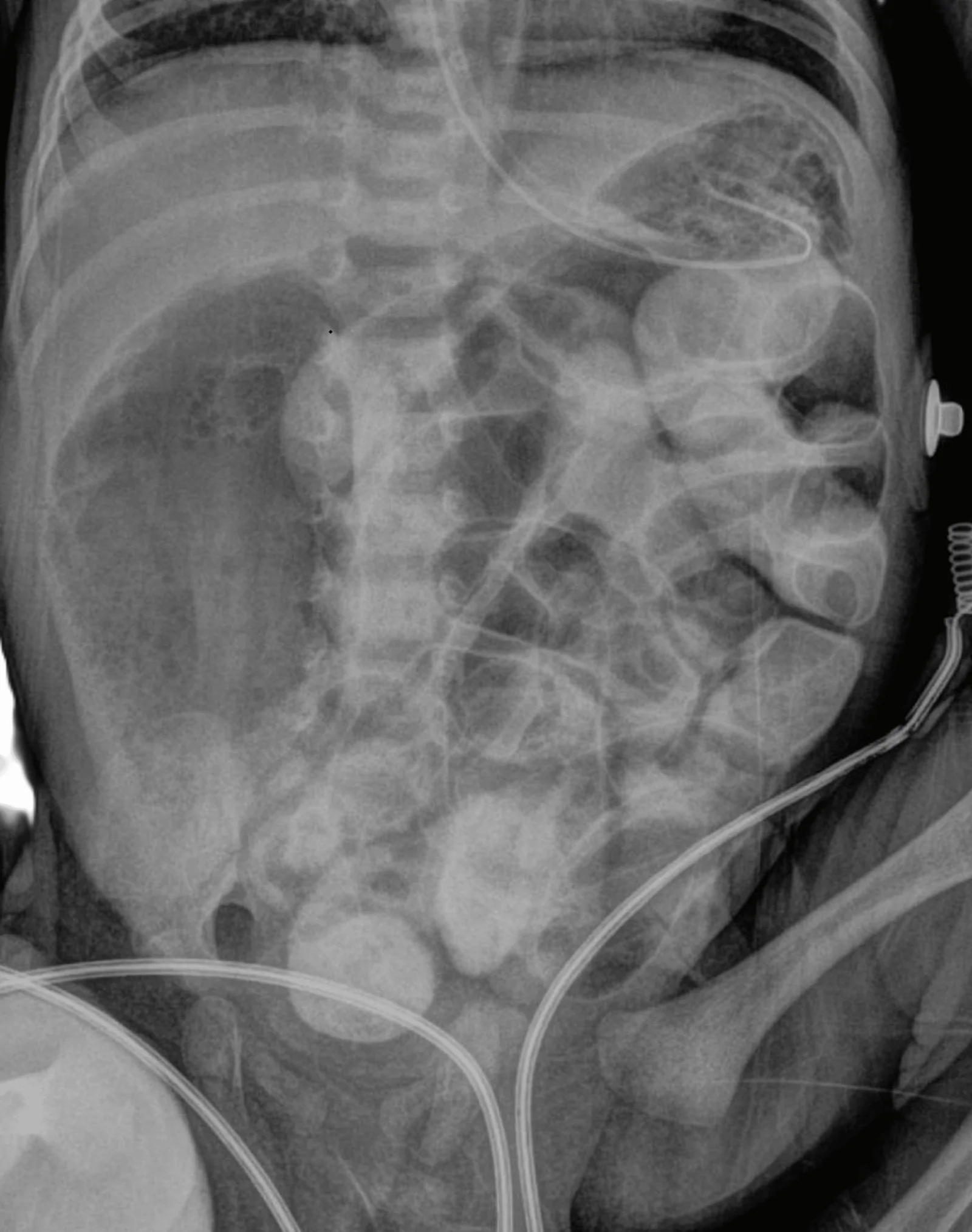
Preoperative oral contrast study Abdominal radiograph obtained after administration of oral contrast through an orogastric tube, showing passage of contrast into the small bowel without opacification of the colon. The tip of the orogastric tube projects over the stomach.

Abdominal ultrasonography showed marked bowel-loop distension and interloop free fluid, without solid-organ abnormalities, masses, or intra-abdominal collections. An oral contrast study administered through an orogastric tube demonstrated passage of contrast into the small bowel without opacification of the colon on the obtained images, accompanied by persistent bowel-loop distension (Figure 2).

Although the study did not establish the precise level or cause of the obstruction, the findings supported the presence of a distal obstructive process. Given the persistent failure to pass meconium, bilious vomiting, abdominal distension, and radiographic evidence of intestinal obstruction, exploratory laparotomy was performed.

Preoperatively, the neonate was kept nil per os, and an orogastric tube was placed for free drainage. A 5-mL intravenous bolus of 10% dextrose was administered, followed by 5% dextrose maintenance fluid supplemented with sodium and potassium at 12 mL/h (123 mL/kg/day; glucose infusion rate, 4.1 mg/kg/min). Glucose and electrolyte levels were monitored, and exploratory laparotomy was performed once the patient was clinically and hemodynamically stable.

**Figure 3:**
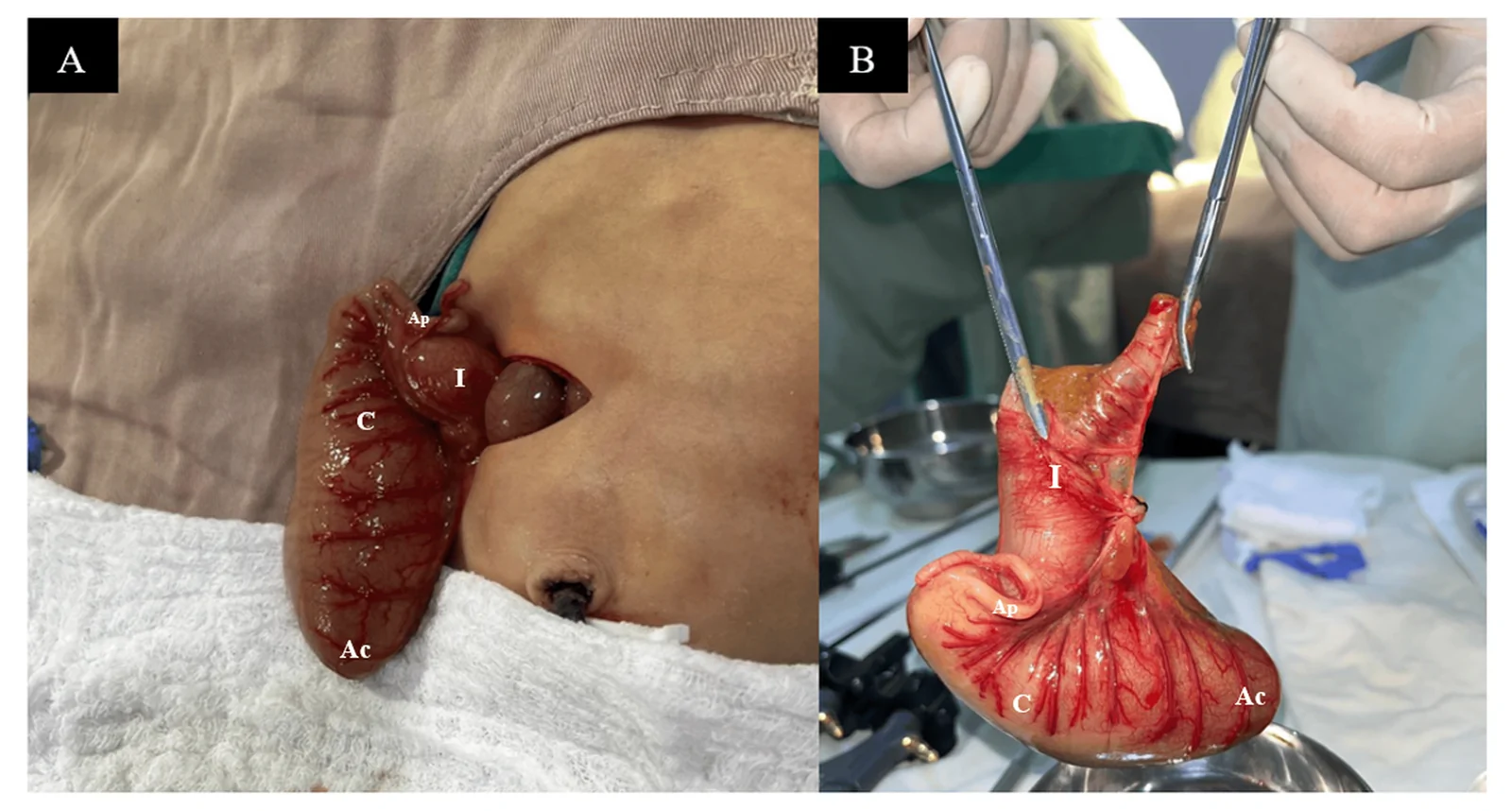
Intraoperative anatomy and resected specimen (A) Intraoperative view showing the terminal ileum (I), cecum (C) with appendix (Ap), and ascending colon (AC), which terminates in a proximal blind-ending pouch at the expected location of the transverse colon. (B) Resected specimen after right hemicolectomy, including the terminal ileum, cecum with appendix, ascending colon, and proximal blind-ending colonic segment.

Exploratory laparotomy revealed atresia of the transverse colon (Grosfeld type IIIa). The ascending colon terminated in a proximal blind-ending pouch at the expected location of the transverse colon, and no continuous transverse colon was identified. The proximal and distal blind-ending segments were completely separated by a V-shaped mesenteric defect. A marked caliber discrepancy was observed between the ascending colon, which measured approximately 30 mm in diameter, and the atrophic descending colon, which measured approximately 10 mm, corresponding to a proximal-to-distal colonic diameter ratio of approximately 3:1. A right hemicolectomy was performed, including resection of the terminal ileum, cecum with appendix, ascending colon, and proximal blind-ending colonic segment (Figure 3).

**Figure 4:**
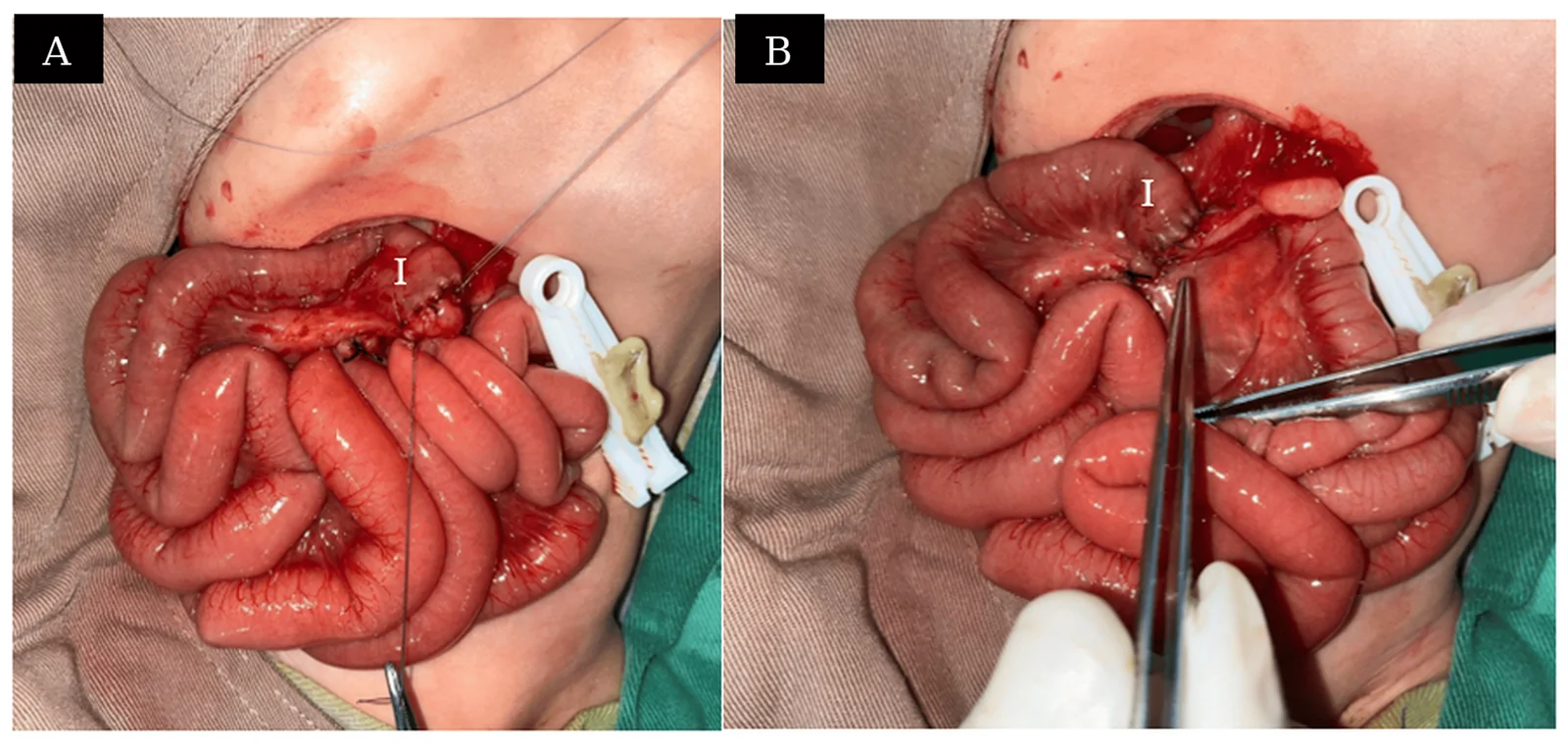
Restoration of bowel continuity after right hemicolectomy Panels A and B show the completed hand-sewn end-to-end anastomosis between the terminal ileum (I) and the descending colon, with approximation of the bowel ends and completion of the anastomotic line.

Bowel continuity was restored through a hand-sewn end-to-end anastomosis between the terminal ileum and the descending colon after gentle dilatation of the distal colonic segment and confirmation of distal patency (Figure 4).

Histopathological examination confirmed colonic atresia, demonstrating interruption of the intestinal lumen with a blind-ending colonic segment. The bowel wall architecture was preserved, without evidence of necrosis. Ganglion cells were identified in the examined proximal colonic specimen. No biopsy of the distal colon was obtained. 

Postoperative course and follow-up

The immediate postoperative course required management in the neonatal intensive care unit with bowel rest, total parenteral nutrition, close clinical monitoring, and antibiotic therapy for late-onset neonatal sepsis. Inflammatory markers were markedly elevated, with a C-reactive protein level of 34.67 mg/dL (commonly used neonatal upper reference threshold: <1.0 mg/dL) and a procalcitonin level of 11,79 ng/mL (reference value after 72 hours of life: <0.1 ng/mL), supporting a marked systemic inflammatory response and a high likelihood of bacterial infection. A molecular sepsis panel detected Enterobacter cloacae, whereas conventional blood cultures remained negative. In the absence of another identifiable infectious focus, the presumed source of sepsis was bacterial translocation across compromised intestinal mucosa secondary to prolonged obstruction, marked bowel distension, and possible mucosal ischemia. The patient completed a seven-day course of cefepime. No anastomotic leak, intra-abdominal collection, surgical site infection, or other intra-abdominal infectious focus was identified. Cerebrospinal fluid analysis and culture were negative. 

The patient showed progressive clinical improvement, with return of bowel function and gradual tolerance of enteral feeding. After two weeks of intensive care, he was transferred to the general ward. He subsequently achieved full enteral feeding with breast milk and was discharged in good general condition 21 days after admission. At the three-month follow-up, he remained asymptomatic, without recurrent obstructive symptoms or postoperative complications.

Long-term follow-up will include serial assessment of stooling patterns, abdominal distension, feeding tolerance, vomiting, growth, and episodes suggestive of enterocolitis. Persistent severe constipation, recurrent abdominal distension or bilious vomiting, poor weight gain, or suspected enterocolitis will prompt pediatric surgical reassessment and targeted evaluation for Hirschsprung disease, including contrast imaging and confirmatory rectal biopsy when clinically indicated.

## Discussion

Colonic atresia is among the rarest congenital gastrointestinal atresias. Tahkola et al. reported 180 patients treated for congenital intestinal atresia between 1947 and 2019, of whom only 24 had colonic atresia [4]. In a population-based Finnish study, the prevalence of colonic atresia was 0.36 per 10,000 live births, confirming its rarity at the national registry level [2]. Involvement of the transverse colon is particularly uncommon and has been described mainly in isolated case reports or mixed series, limiting the evidence available for this specific anatomic location [5].

Cabrera Valerio et al. reported a six-case series comprising three female and three male neonates diagnosed between 24 and 84 hours after birth. All atresias involved the right hemicolon. The main clinical manifestations were abdominal distension, failure to pass stool, and bilious or feculent vomiting. Surgical management included both primary anastomosis and staged repair, and one patient diagnosed at 72 hours of life died [6]. However, the small number of patients does not allow an association between diagnostic timing and mortality to be established. In the present case, the obstructive symptoms had been present since birth, but the mother first sought medical attention when the patient was seven days old. Therefore, the diagnosis at seven days reflects delayed presentation to medical care rather than late onset of the disease or an in-hospital diagnostic delay. This emphasizes the importance of advising caregivers to seek prompt medical evaluation when a neonate fails to pass stool or develops progressive abdominal distension and bilious vomiting.

The most widely accepted pathophysiologic mechanism for jejunoileal and colonic atresia is an intrauterine vascular insult resulting in segmental ischemia, necrosis, and resorption of the affected bowel. This process produces blind-ending intestinal segments and may be accompanied by a mesenteric defect. Proposed causes of the vascular disruption include intrauterine volvulus, fetal intussusception, internal herniation or strangulation, and thromboembolic events. Although the precipitating event is rarely demonstrated, this mechanism is consistent with the anatomic findings observed in type IIIa atresia [7].

Plain abdominal radiography is the initial imaging modality in neonates with suspected intestinal obstruction. Typical findings include dilated bowel loops and, in distal obstruction, a relative paucity of gas in the rectum and distal bowel [8,9]. When colonic atresia is suspected, contrast enema is commonly used to characterize the unused distal colon and may demonstrate a microcolon with an abrupt cutoff at the level of the atresia [10,11].

In the present case, abdominal radiography demonstrated diffuse bowel-loop dilatation and relative paucity of distal gas. An orally administered contrast study performed through an orogastric tube showed progression of contrast into the small bowel without opacification of the colon on the obtained images. This finding was compatible with a distal intestinal obstruction but could neither establish the diagnosis of colonic atresia nor accurately localize the obstruction to the transverse colon. Operative exploration was prioritized because of the persistent complete obstruction since birth, bilious vomiting, abdominal distension, tachycardia, a 15.5% decrease from birth weight, and ultrasonographic evidence of interloop free fluid. Because a contrast enema was not performed, the unused distal colon was not characterized preoperatively; this represents a limitation of the diagnostic evaluation.

The Grosfeld classification provides a standardized description of the anatomic patterns of colonic atresia. In the series by Saha et al., type IIIa was the most frequent subtype, occurring in six of nine patients, and the transverse colon was the most commonly affected segment, accounting for five cases [12]. El-Asmar et al. also reported a predominance of type IIIa atresia, identified in six of 12 patients, followed by type II in four patients and types I and IV in one patient each [13]. Although these series are small, they suggest that type IIIa is a frequently encountered morphologic pattern. The present case was classified as Grosfeld type IIIa: the ascending colon ended in a proximal blind pouch at the expected location of the transverse colon, the transverse colonic segment was absent, and the proximal and distal blind ends were separated by a V-shaped mesenteric defect.

Surgical management varies between primary anastomosis and staged repair with initial enterostomy. The choice depends on the neonate’s clinical condition, the viability and caliber of the bowel segments, the degree of size discrepancy, the presence of perforation or contamination, associated intestinal abnormalities, and the possibility of concomitant Hirschsprung disease. In a recent multicenter study, Patwardhan et al. identified concurrent Hirschsprung disease in eight patients (9.5%), seven of whom were initially managed with diversion. Overall, 58 patients (69%) underwent initial diversion. Patients treated with primary anastomosis required fewer operations but received more days of parenteral nutrition, whereas the other evaluated outcomes did not differ significantly [14]. These findings support individualized surgical decision-making rather than the routine selection of either strategy. In a broader retrospective cohort of 92 neonates with intestinal atresia, Hillyer et al. found that primary anastomosis was associated with a shorter hospital stay than staged repair (27 versus 95 days) and fewer days of total parenteral nutrition (19 versus 74.5 days) [15]. However, that study included different sites of intestinal atresia and its findings cannot be directly extrapolated to colonic atresia, particularly when distal aganglionosis has not been excluded.

In the present patient, primary anastomosis was selected after right hemicolectomy because the remaining bowel appeared viable, distal patency was confirmed intraoperatively, and the descending colon could be gently dilated to permit an end-to-end ileocolic anastomosis. However, no biopsy was obtained from the distal colon or rectum. An important limitation of our center is the lack of intraoperative frozen-section histopathology, which prevented the assessment of distal ganglion cells and the exclusion of congenital aganglionic megacolon (Hirschsprung disease) before primary reconstruction. Ganglion cells were identified only in the resected proximal colonic specimen; therefore, this finding does not exclude aganglionosis of the distal colon or rectum [16]. The absence of recurrent obstructive symptoms and the presence of normal bowel function at three-month follow-up are reassuring but do not definitively exclude associated Hirschsprung disease. The lack of distal histologic assessment and the relatively short follow-up period should consequently be acknowledged as limitations. The favorable postoperative outcome demonstrates the feasibility of primary anastomosis in this selected patient but does not establish its superiority over staged repair.

Outcomes in colonic atresia are influenced by associated congenital anomalies, anatomic complexity, delays in diagnosis, and the need for multiple procedures. In the Finnish population-based study by Kankaristo et al., only 41.7% of cases were isolated, and 50% had multiple congenital anomalies; nevertheless, overall survival was 97% [2]. Tahkola et al. similarly reported associated malformations in approximately half of patients with intestinal atresia and documented substantial improvement in survival over time [4]. These findings indicate that prognosis is generally favorable when the atresia is surgically correctable, major associated anomalies are absent, and specialized neonatal support is available.

This case is noteworthy because of the unusual transverse colonic location and type IIIa morphology. Although obstructive symptoms were present from birth, the diagnosis was established at seven days of life because the mother first sought medical attention at that time. The case also highlights the need to consider distal aganglionosis when primary reconstruction is planned, particularly in centers where intraoperative frozen-section biopsy is unavailable.

## Conclusions

Transverse colonic atresia is a rare cause of neonatal distal intestinal obstruction. It should be considered in neonates with failure to pass stool, bilious vomiting, and abdominal distension, even when medical evaluation occurs beyond the first days of life. Surgical management should be individualized according to the patient’s clinical status, bowel anatomy, distal patency, and intraoperative findings, with assessment for distal aganglionosis before primary reconstruction whenever possible. When intraoperative frozen-section biopsy is unavailable, this limitation should be acknowledged, and close postoperative follow-up is essential.
